# Data integration strategies for whole-cell modeling

**DOI:** 10.1093/femsyr/foae011

**Published:** 2024-03-27

**Authors:** Katja Tummler, Edda Klipp

**Affiliations:** Humboldt-Universität zu Berlin, Faculty of Life Sciences, Institute of Biology, Theoretical Biophysics,, Invalidenstr. 42, 10115 Berlin, Germany; Humboldt-Universität zu Berlin, Faculty of Life Sciences, Institute of Biology, Theoretical Biophysics,, Invalidenstr. 42, 10115 Berlin, Germany

**Keywords:** complex networks, yeast cell model, emerging standards, single cell

## Abstract

Data makes the world go round—and high quality data is a prerequisite for precise models, especially for whole-cell models (WCM). Data for WCM must be reusable, contain information about the exact experimental background, and should—in its entirety—cover all relevant processes in the cell. Here, we review basic requirements to data for WCM and strategies how to combine them. As a species-specific resource, we introduce the Yeast Cell Model Data Base (YCMDB) to illustrate requirements and solutions. We discuss recent standards for data as well as for computational models including the modeling process as data to be reported. We outline strategies for constructions of WCM despite their inherent complexity.

## Teaser

Whole-cell models are useful to understand cellular life and replication in their complexity, but are challenging with respect to their need in data and modeling efforts.

## Introduction

Whole-cell models (WCM) have proven to be as insightful as they are challenging to establish. By covering systemic effects and interactions between individual biological processes, they pave the way to a deeper mechanistic understanding of cells, organisms, and diseases.

As for all models, WCMs rely on experimental data that are ideally both high resolution in time and space and high quality in terms of reproducibility and accuracy. However, due to the wide variety of biological processes covered, the data sets on which WCM are based have to fulfill further requirements: They have to be obtained from comparable—ideally the same—experimental conditions for all measurement methods of diverse biomolecules and processes. They have to cover all relevant processes in a quantitative manner. Naturally, they have highest information content if they are acquired from single cells or at least cell cycle synchronized colonies.

Due to the technical complexity of modeling all processes in a cell, WCM is so far carried out for simple model organisms only.

WCM approaches are available, e.g. for *Mycoplasma genitalium*, a microorganism with a well-characterized small genome. Also, for the eukaryotic model organism *Saccharomyces cerevisiae* WCM efforts have been established, harnessed with decades worth of experimental data available from literature. This data has, however, been obtained from many different strains, culture conditions, and measurement methods, resulting in questionable comparability of the gained results. On the other hand, if all environmental conditions are carefully considered, the data can be compiled and converted into a fully quantitative description of a prototypic yeast cell.

Here, we discuss the types of data needed for a yeast WCM and introduce a database that contains a systematic collection of data intended to parameterize such a model. We review existing models for parts of the cellular life as well as strategies for their integration.

The Yeast Cell Model Data Base (YCMDB) is available online (https://www.tbp-klipp.science/ycmdb/).

### What is a whole-cell model?

A whole-cell model is a computational model that attempts to simulate the behavior of an entire living cell, capturing the intricate interactions between various cellular components such as proteins, metabolites, genes, and regulatory networks. These models aim to provide a comprehensive understanding of cellular processes at the systems level, allowing researchers to study how different molecular components work together to carry out functions such as metabolism, signaling, and gene expression.

WCM typically integrate experimental data from various sources, including genomics, proteomics, metabolomics, and bioinformatics databases, to construct a detailed representation of cellular processes. They often utilize mathematical and computational techniques such as differential equations, stochastic modeling, and constraint-based modeling to simulate the dynamics of cellular systems under different conditions.

The development of WCM holds promise for advancing our understanding of cellular biology, enabling the prediction of cellular behaviors in response to perturbation and providing insights into the underlying mechanism of diseases. These models can also be used to guide experimental research and facilitate the design of novel therapies and biotechnological applications.

There is now a series of models available that strive for a holistic description of cellular processes. However, they are different in nature and, therefore, require different types of data. Most of these models represent different bacteria, as processes in prokaryotic cells are less complex, especially due to less compartmentalization. We start with a brief introduction to some WCM efforts.

The E-Cell project was launched at Keio University by the Tomita group already in 1996 and studied *M. genitalium*. This organism is especially interesting to build a WCM, because it has the smallest known genome of all free-living organisms with 580 kb coding for 563 genes (Fraser et al. 1995). The small size of the genome gave rise to the assumption of a minimal functional gene set with only essential genes and a unique assignment of genes and functions. The E-Cell project intended to develop general technologies and theoretical support for computational biology with the grand aim to make precise whole cell simulations at the molecular level possible. Their first virtual hypothetical cell published in 1997 and 1999 contained 127 genes, was self-sustained by producing energy from glucose with the help of the encoded genes, but did comprise neither proliferation, DNA replication nor cell cycle (Tomita et al. 1997, 1999). The endeavor continues as E-Cell Project (e-cell.org). The whole-cell model of Karr, Covert and colleagues intended to predict phenotype from genotype, again for *M. genitalium* (Karr et al. 2012). It contains 28 submodels of cellular processes and 16 variables integrate cellular functions. Shuler and colleagues developed a series of minimal models for *E. coli*, which included growth on glucose, certain synthesis pathways as well as cell size and cell shape (Domach et al. 2000, Castellanos et al. 2004). Morgan and colleagues introduced in a series of publications a framework of whole-cell modeling that extended towards growth and division (Morgan et al. 2004, Surovtsev et al. 2008, 2009).

Recently, an alternative type of models used the principle of resource balance analysis to quantitatively predict whole cell properties such as growth rate, metabolic fluxes, or abundances of molecular machines in different bacteria (e.g. Goelzer et al. 2015, Weisse et al. 2015, Bulovic et al. 2019). Molenaar et al. (2009) used a very simplified representation of a cell to elaborate the hypothesis that growth strategies are the result of tradeoffs in the economy of the cell, in which growth rate maximization of the entire system is the objective, and where the only limitations are those set by the laws of physics and chemistry. Elsemman et al. (2022) applied comparable principles to yeast to analyze metabolic adaptation to nutrient conditions. The fate of single cells, their cell cycle, shape, or diversity are not considered in this type of modeling approach.

**Figure 1.**
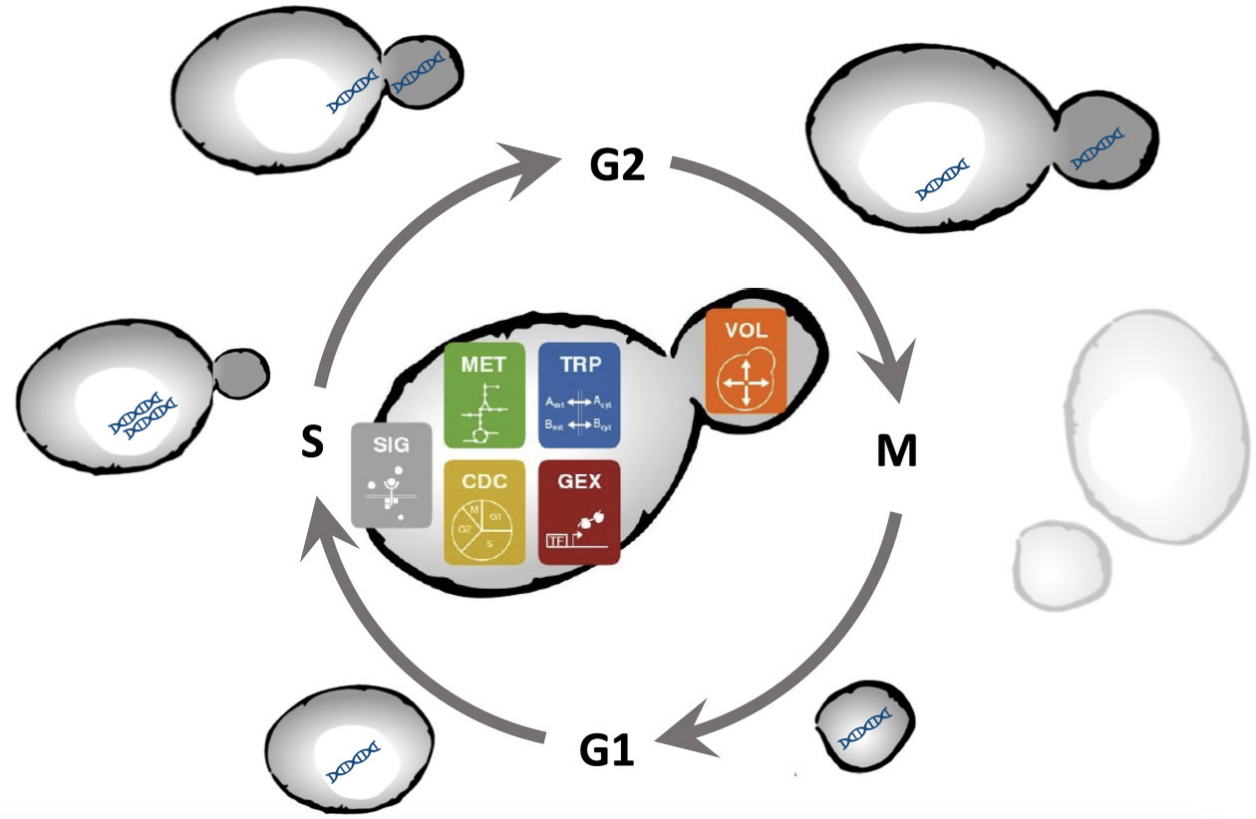
A WCM captures cellular maintenance and the cell division cycle, as well as cellular responses to external stresses. Here, icons within the sketched yeast cell symbolize the major processes and MET stands for metabolism, SIG for signaling, GEX for gene expression, TRP for transport, CDC for cell division cycle, and VOL for volume changes and growth. Around the cell, G1, S, M, and G2 indicate the cell cycle phases, where the cell starts as a small cell with only one copy of DNA and then grows in G1 phase. In S phase, DNA is duplicated and yeast cells start to form a bud. After another phase of growth in G2, cells organize division in mother and daughter cell during M phase.

Our understanding of a whole-cell model as relevant for the considerations below comprises three major features (see Fig. 1). First, it is focused on a single, though average, cell. Second, it is dynamic, which means it covers the life of that cell from one division to the next division. Third, it should include all major processes of cellular life along with the machinery necessary for cellular replication, though the level of detail can vary. An additional desired feature is responsiveness of the cell to selected external changes of conditions and all kinds of cues.

**Figure 2.**
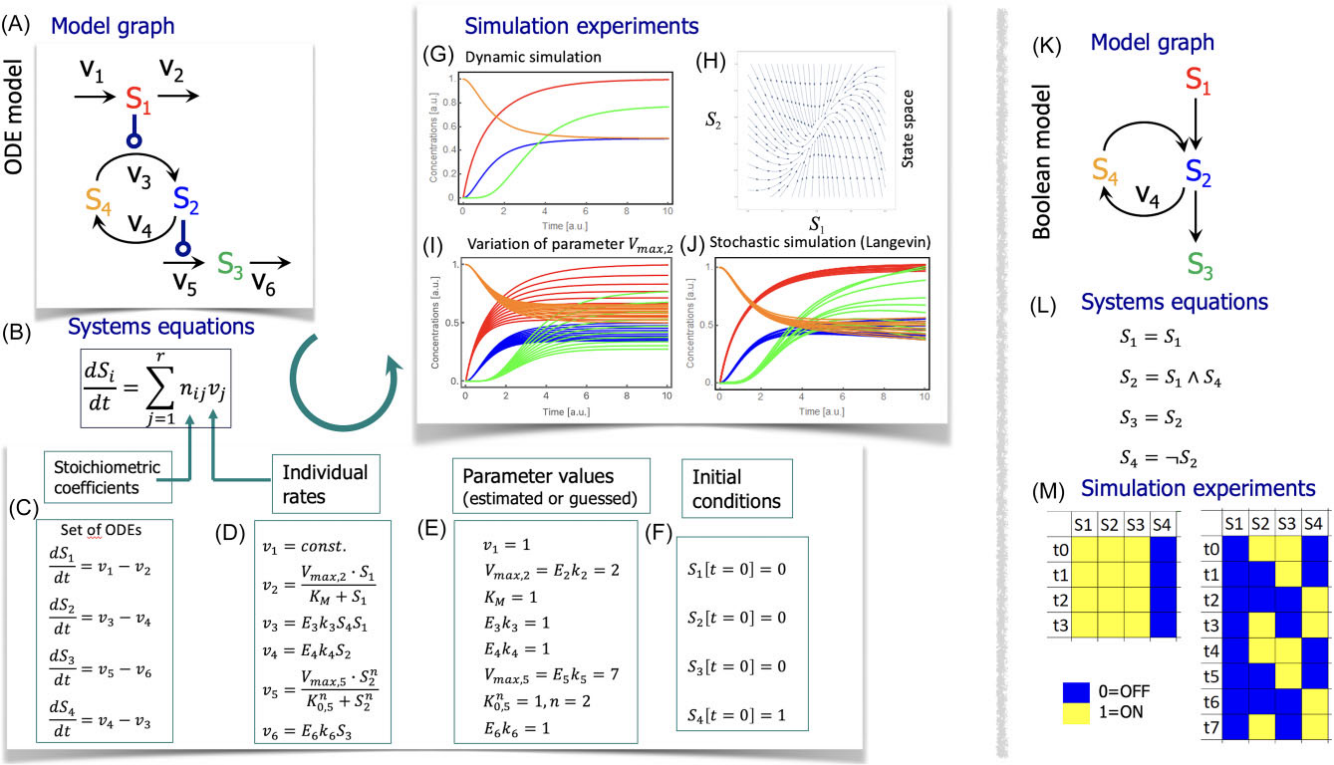
Overview over typical steps in mechanistic modeling, illustrating both ODE modeling (A)–(J) and Boolean modeling (K) and (L). (A) The information about the processes to be covered by the model can be given in graphical representation. The example used here can be representative both for metabolic or signaling processes: compound *S*_1_ is produced and degraded by reactions 1 and 2 (with velocities *v*_1_ and *v*_2_), compounds *S*_2_ and *S*_4_ are converted into each other by reactions 3 and 4, compound *S*_3_ is also produced and degraded by reactions 5 and 6. Compounds *S*_1_ and *S*_3_ modify (activate or inhibit) the velocities of reactions 3 and 5, respectively, without being consumed or produced themselves by these reactions. (B) The systems equations, in general, represent the temporal changes of the compounds *S_i_* (denoted by the time derivative *d*/*dt*), which is given by the rates (or velocities) *v_j_* combined with the stoichiometric coefficients. The necessary steps such that the system can be simulated are sketched in panels (C)–(F): (C) represents the set of systems equations for the example in (A). (D) illustrates choices for rate expressions. *v*_3_, *v*_4_, and *v*_6_ follow mass action where parameters *k* stand for rate constants, *v*_2_ is an example for Michaelis–Menten kinetics (with *V*_max_ maximal velocity and *K_M_* Michaelis constant) and *v*_5_ for Hill kinetics (*K*_0,5_ is the concentration giving half maximal velocity, *n* is the Hill coefficient). (E) Parameter values can be either obtained from databases, estimated from experimental data (genomics, proteomics, metabolomics, and biophysical measurements) or simply guessed (as done here). Briefly, parameter estimation requires systematic repeated simulation with different parameter values and comparison with experimental data with the aim to minimize the difference between data and simulation. (F) For a simulation to start, one has to determine the initial conditions. (G)–(J) are examples for simulation experiments based on the ODE system in panels (C)–(F). (G) shows a time course simulation. (H) presents the state space for *S*_1_ and *S*_2_ were vectors indicate the direction of motion from different starting points. (G) and (H) show that the system moves toward a steady state. (I) A typical way to analyze the ODE system is sensitivity analysis, i.e. testing the effect of small parameter variations on the dynamics. Here, parameter *V*_max2_ has been varied (10 simulations with different values). (J) shows the result of a stochastic simulation of the same system with the Langevin approach, where a noise term is added to each equation resulting in slightly different dynamics for each of, here, 10 simulations. (K)–(M) Boolean model of a comparable system: Component *S*_1_ activates *S*_2_, *S*_2_, and *S*_4_ can be converted into each other (thereby annihilating the other component) and *S*_2_ activates *S*_3_. Here, all compounds can have only two states, ON or OFF; also time proceeds in discrete steps. (K) Graph of the model. (L) Systems equations denote the state of the compound at the right side at time *t* + 1 as function of the state of components at the left side at time *t*. These changes are expressed with Boolean rules. (M) shows two simulation experiments with different initial conditions, where *S*_4_ starts ON at *t*_0_ in both cases and *S*_1_ is either OFF or ON. If *S*_1_ is OFF, the system is already at a fixed point and shows no changes in the following time steps. If *S*_1_ is ON, the system oscillates, i.e. it has a cyclic attractor. For both ODE and Boolean modeling, it is often necessary to revise the model and repeat the modeling steps, i.e. network creation (components and reactions), assignment of rate expression or rules, and the parameter values, until the model behavior correctly reflects the experimentally observed behavior of the system.

In order to give a reader who is not experienced with mechanistic modeling a brief introduction into the steps of model development and requirements, we illustrate the workflow of dynamic modeling of a small biochemical reaction network in Fig. 2.

### Standardization of data generation, storage, and sharing

The FAIR (findable, accessible, interoperable, and reusable) principles have become indispensable for any project that uses shared data or intend its data to be reused (Wilkinson et al. 2016, David et al. 2023). Well-curated databases such as GenBank, PDB, UniProt, CheBi, Ensemble, or Pfam are standard resources for data in modeling projects (Benson et al. 2012, Hastings et al. 2016, Burley et al. 2017, Mistry et al. 2021, Martin et al. 2023, Uniprot et al. 2023). However, other types of data are useful for or required to parameterize a WCM, which cannot be found in the standardized databases.

The modeling process itself establishes a type of data critical for WCM. Any computational model is characterized by a series of choices or decisions: (i) the assignment between real-world modalities (e.g. abundances, volumes, pressure, and temperature) and variables, parameters or constants of the model, (ii) the treatment variables as discrete (e.g. molecule numbers) or continuous (concentrations), (iii) the discrimination between what belongs to the model and what to its environment, (iv) the types of interactions between the variables, i.e. whether there is an interaction and which model describes this interaction best (e.g. either mass action of Michaelis–Menten kinetics or another kinetic type for metabolic interconversions), (iv) the formalism to update the state of variables, e.g. set of differential equations or Boolean model or stochastic simulation, including its detailed settings (e.g. which program and which solver to use). Moreover, each computational experiment with the model creates a data set, i.e. which model perturbation (e.g. parameter change or addition of a cue or set of repetitions for stochastic models) and type of simulation (e.g. steady state or time course) has been performed and what is the outcome. Thus, the model formalism and all relevant decisions have to be reported in sufficient detail to reproduce the model results. This also includes implicit assumptions. In addition, the parameter estimation process or alternative ways to obtain parameters have to be outlined. More and more computational projects are organized in shareable repositories such as Git, which makes this kind of information accessible to the scientific community.

In the last 20 years, the scientific area of mathematical modeling in biology (often called systems biology) has seen an explosion of standards that have been developed for different purposes and modalities, starting with the systems biology markup language (SBML) standard on the modeling site (Hucka et al. 2003) and minimum information requested in the annotation of biochemical models (MIRIAM) (Novere et al. 2005) on the experimental side. The SBML is a standard format used for representing computational models of biological processes in systems biology. It provides a means for describing models in a standardized, machine-readable format, enabling interoperability between different software tools and facilitating model sharing and collaboration among researchers. SBML defines a set of rules and structures for representing biochemical reaction networks, including information such as species, reactions, compartments, and parameters. It is designed to be human-readable as well as machine-readable, making it accessible to both researchers and software applications. SBML allows modelers to describe complex biological systems in a concise and unambiguous way, facilitating the exchange of models between different modeling and simulation tools. This standardization helps promote reproducibility and transparency in systems biology research by enabling researchers to share their models and results more easily. The most recent version of SBML is SMBL Level 3 offered as extensible format for exchange and reuse of biological models (Keating et al. 2020). MIRIAM is a set of guidelines and recommendations for annotating computational models in systems biology. It was developed to improve the clarity, consistency, and interoperability of model annotations, facilitating the exchange and reuse of models among researchers and software tools. It provides a framework for annotating models with metadata that describe various aspects of the model, such as its creators, version history, biological context, and experimental conditions. This metadata helps ensure that models are properly documented and can be understood and used by others. The COMBINE standards (https://co.mbine.org/) comprise a series of modeling formats, i.e. next to SBML also BioPax (Demir et al. 2010) as a language to integrate, analyze, and exchange pathway data, CellML (Clerx et al. 2020) to store and exchange computer-based mathematical models, Synthetic Biology Open Language Data (SBOL Data, Mclaughlin et al. 2020) for the description and the exchange of synthetic biological parts, devices and systems. The Systems Biology Graphical Notation (SBGN; Novere et al. 2009) is a standardized graphical language used to represent biological processes, pathways, and networks. It provides a set of symbols and rules for creating visual representations of biological systems, enabling researchers to communicate complex biological concepts in a clear and unambiguous way. SBGN consists of three main languages or branches, i.e. the Process Description focuses on the relationships between biological entities and processes within a network, the Entity Relationship emphasizing the relationships between biological entities, such as proteins, genes, and complexes, without specifying the exact nature of the interactions, and the Activity Flow (AF) describing the flow of biological activities or signals within a network. Last not least, The Simulation Experiment Description Markup Language (SED-ML; Bergmann 2011) is a standard format for describing computational simulation experiments. It provides a means for specifying the setup, execution, and postprocessing of simulation experiments in a machine-readable format, facilitating the reproducibility and exchange of simulation studies among researchers and software tools. It includes model description, simulation settings, experimental conditions, as well as specifications of outputs and analyses.

There are open standard model repositories such as BioModels (Malik-Sheriff et al. 2020, Glont et al. 2018), CellCollective (Helikar et al. 2012), JWSonline (Olivier and Snoep 2004), and Physiome (Hunter 2004), where models can be stored, accessed and in some cases even created for a specific purpose.

Specific communities develop not only their own standards, but even their related test tools to assess the quality of models in that field. A recent example is metabolic model tests for genome-scale metabolic models (Lieven et al. 2020).

### Data integration entails, but is more than storing data in the same database

What is data when we talk about whole-cell modeling? First of all, it comprises all results of experimental investigations of the system to be modeled. Second, it entails all so-called meta-data, i.e. extended information about these experiments—such as cultivation context, growth rates, or instruments used—i.e. helpful or necessary to correctly interpret the results. And finally, it has to cover all the aspects of the modeling exercise itself. We will elucidate the experimental part of these three types of data below.

Within the realm of a WCM, the experimental data should preferentially comprise comprehensive information about all parts and processes of a living cell. It typically includes compound abundance information such as genomics, transcriptomics, proteomics, phosphoproteomics, and metabolomics. It leads to values of abundances or relative abundance changes of proteins, their complexes and modification states (e.g. phosphorylation and methylation), abundances of mRNAs, and concentrations of metabolites or changes thereof. In the best case, this data is time-resolved over relevant processes such as cell cycle or the duration of a response to a dedicated perturbation. Further information is required on rates, such as growth rates, rates of nutrient uptake and release of waste or other components, respiration rates, reaction rates, rates for transcription and translation, and rates for decay of cellular compounds. Relevant biophysical information comprises cell mass, volume, shape, surface area, sizes of compartments, external and internal osmolarity as well as turgor pressure.

Meta-data is required to, first, make different data sets comparable and, second, characterize the context of the measurement. It includes all information about the analyzed organism (e.g. species, strain, accession, and genetic modifications). The experiment itself must be sufficiently described: what has been measured under which circumstances, which instruments have been used, maybe even specific settings of the instrument, if relevant. Next, different types of physical information must be reported, such as temperature, pH, and medium composition. And finally, the changes in the environment during the experiment can be relevant, e.g. which nutrients were depleted, which compounds were released by the investigated organism. This information is crucial to understand whether the measured values are comparable or convertible between different studies. Only if, i.e. possible, they can be used in the same mathematical model. As an example, the pH value of the medium has an effect on many cellular processes—internal metabolite concentrations, membrane potentials, respiratory rates, and more. Hence, data acquired in media with vastly different pH values cannot be used without at least precaution or recalculations in the same model.

### The YCMDB as example for systematic collection and integration of data

**Figure 3.**
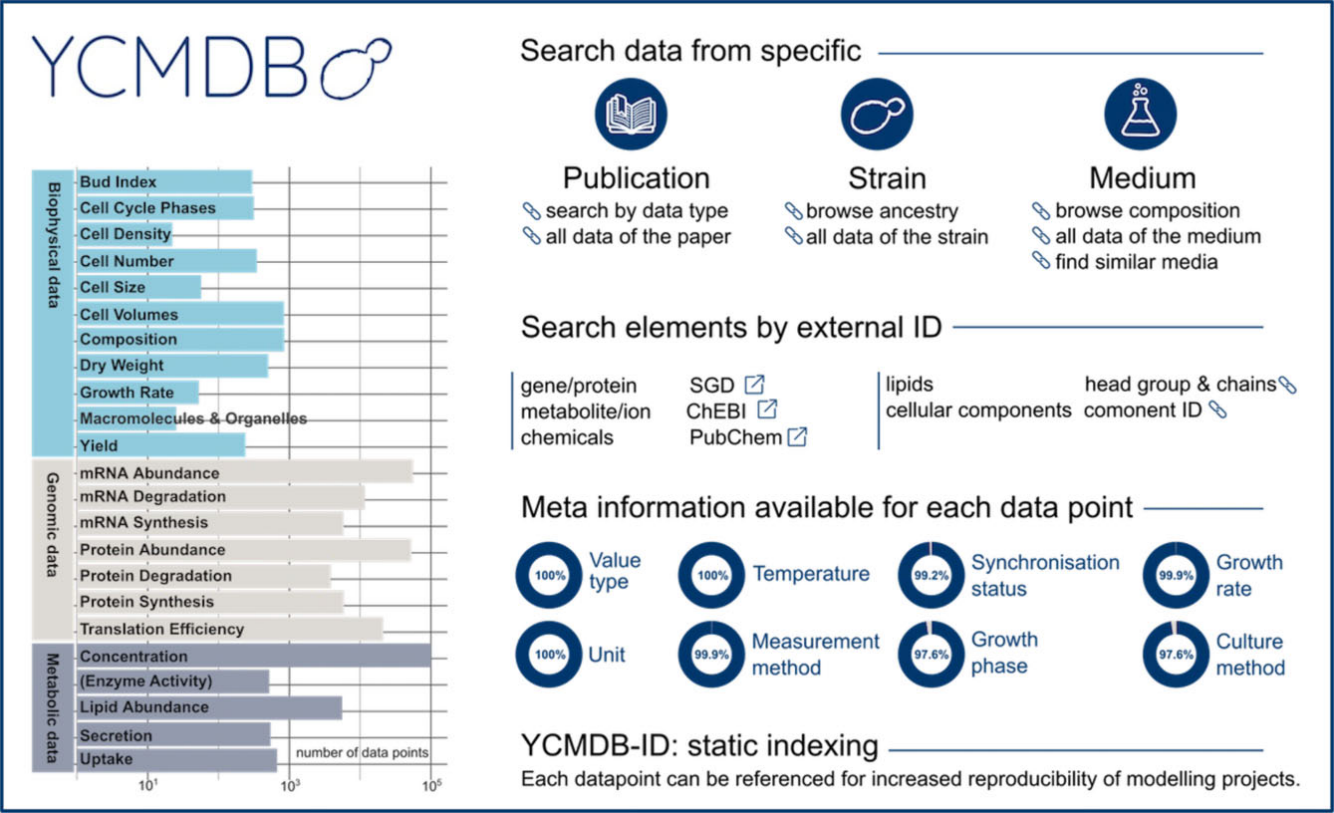
Overview of the contents and search functionalities of the YCMDB.

Making data reusable as well as understanding the exact experimental background and, hence, the data usability for a specific modeling project is the idea behind YCMDB—a curated database for quantitative modeling of yeast (https://tbp-klipp.science/ycmdb). An overview of the database is represented in Fig. 3. The database collects published as well as novel data that fulfills the requirements for insightful mathematical models: quantitativeness (including measurement uncertainties), ideally temporal and single-cell resolution as well as a high level of annotation. Data on compound concentrations and uptake rates, synthesis, and degradation rates as well as general biophysical numbers such as cell size or cell cycle phase lengths are included in the relational DB.

The number of data points contained in the YCMDB is 10 727 for metabolic data, 151 299 for genomic data, and 3488 for biophysical data. They stem from 74 publications. A total of 164 different media have been reported as well as 63 different strains (excluding k.o. libraries). A total of 16.3% of the contained data belongs to BY4742 and 73.5% to CEN-PK.

Each data point is annotated with specific meta-information to apprise how comparable the data is to the modeled scenario (culture conditions, growth phase, and measurement techniques) and referenceable with a static ID. The high degree of interconnectivity between the datasets allows to attain additional information for unit conversions and adaptation to e.g. different cell sizes as well as to find data points stemming from similar experimental setups. YCMDB is an integrative resource for quantitative yeast modeling projects on any scale and fosters reproducible modeling efforts.

### Data collection, annotation, conversion, and interconnection in YCMDB

The majority of data was collected from experimental studies published through the last decades. From each publication, relevant data points were extracted from tables, supplements, figures (WebPlotDigitizer; Rohatgi 2020) and text, followed by careful annotation with relevant meta information:

Publication, strain, medium, unit, culture mode (continuous/batch), growth phase, growth rate, temperature, synchronization status, aeration, measurement method, and a comment usually to ease finding of the data point in the respective publication. If the data is time-resolved, the time point and the unit of time (e.g. s/min/h) are noted. We further note, which type of value the data point represents along with information on the measurement accuracy: The value type can either be an average (with standard deviation), median, minimum/maximum value, or a plain value with no accuracy information. Where available, we track the number of replications.

Data points were considered if they were quantitative (with a convertible unit) and had information about the used strain and culture conditions.

Each datapoint can be referenced unambiguously by a static YCMDB-Id. This way, model calibration efforts can be documented in a reproducible manner, e.g. in combination with PETabs (Schmiester et al. 2021).

Oftentimes, data points with exactly the right conditions will not be available anywhere in the literature. Therefore, YCMDB aims at tightly interconnecting the data such that the search for data points that match a user’s conditions close enough becomes possible along with an appraisal of the differences to be considered. Therefore, the strain ancestry was compiled from literature and is made available in YCMDB (see Fig. S1, Supporting Information). This allows to search for data from similar strains and, if found, to understand which genetic alterations separate the strains.

Data points for similar media can be searched, the medium composition is compiled in detail, for each medium constituent concentrations (if available) are listed and externally referenced via PubChem (Kim et al. 2021). External database IDs are used for unambiguous identification of metabolic compounds (Hastings et al. 2016) and genes/proteins (Cherry et al. 2012).

The database was implemented in MySQL and is available via an online user interface based on R shiny. The code is available via GitLab.

### Application areas of YCMDB

Yeast literature is rich in datasets, with early publications measuring the nuts and bolts of the cell with a variety of techniques. The measurements were tailored to the individual question of the study, using different strains, media, growth conditions, analytic techniques, and displaying results in wild units. To make the available data usable for mathematical modeling and quantitative analysis, effects of these differences in experimental setups have to be accounted for. YCMDB collects relevant data points along with all available meta-information, to allow researchers to (i) quickly and reproducibly find data points required for the calibration of their models and (ii) to assess how suitable and comparable the values are to the modeled scenario.

All data points included in YCMDB are quantitative, ideally also of single cell and temporal resolution. YCMDB contains data for 22 different data types measuring the most important concentrations and biophysical properties of the cells. This includes (i) values for biophysical properties such as size or volume and information about the cellular state such as cell cycle phase, (ii) abundances and rates of change for mRNAs and proteins as well as (iii) metabolic data. The data is highly interconnected and YCMDB allows for cross-functional searching to discover further relevant data points with similar metadata. Data is stored in the original units of the publication, conversion to a desired unit is in many cases possible via the linked information such as cell size, growth rate, etc..

### The YCMDB web interface

We provide a browsable web interface based on R shiny for YCMDB, available on https://tbp-klipp.science/ycmdb.

Each data point that fulfills the requirements gets assigned to a static identifier, the YCMDB-ID. This IDcan be used for reference purposes of the developed models and makes each used data point traceable. The YCMDB ID is displayed at the beginning of each row in the web interface tables.

The three different data classes—metabolic, genomic, and biophysical—re-explorable in separate tabs of YCMDB. Each database entry is linked to an external identifier: ChEBI-IDs for metabolites, SGD-Ids for genes, protein, and mRNAs. We aim to provide concentrations or numbers for cellular components as well as change rates (uptake, secretion, synthesis, degradation, and translation) and characteristic features of the entire cell (density, size, weight, volume, organelle numbers, and duration of cell cycle phases in the tab for biophysical data). Each entry has a numerical value taken directly from the publication along with the given unit, where available a range or standard deviation is included (can be selected in the “select columns to show” drop down menu of each tab). Users can search for data entities in each tab and download the filtered and ordered datasets. A full download of all data is available as well.

Strain ancestry was compiled, including mutations and the literature trail which led to the strains’ development, where possible. The Strain Tab provides a browsable ancestry tree (see Fig. S1, Supporting Information) combining all information along with links to external publications and the data points available from the strain.

Data was only included from experiments that clearly stated the composition of the used media. All compounds are listed and linked to the PubChem database for comparability, the composition of commonly used media bases such as YNB is also resolved to the level of individual constituents. The Medium Tab allows searching for similar media via a graphical clustering representation where medium compounds can be highlighted (similar media appear closer together on the graph, t-Sne clustering). This allows to find useful data points even if nod data for the exact same cultivation conditions is available in YCMDB.

Publication information is available in the Publication Tab allowing users to search for publications that include a specific data type. For each publication a list of further available data points is shown, which is especially important for whole cell modeling as it requires data on all cellular processes being as consistent as possible.

The process of curation data for YCMDB requires a high level of care and accuracy to ensure that the set quality criteria are met for each data point. In the future we plan to add a data submission feature, which will allow the user to upload their own relevant data along with all required meta information.

### Divide and conquer

**Figure 4.**
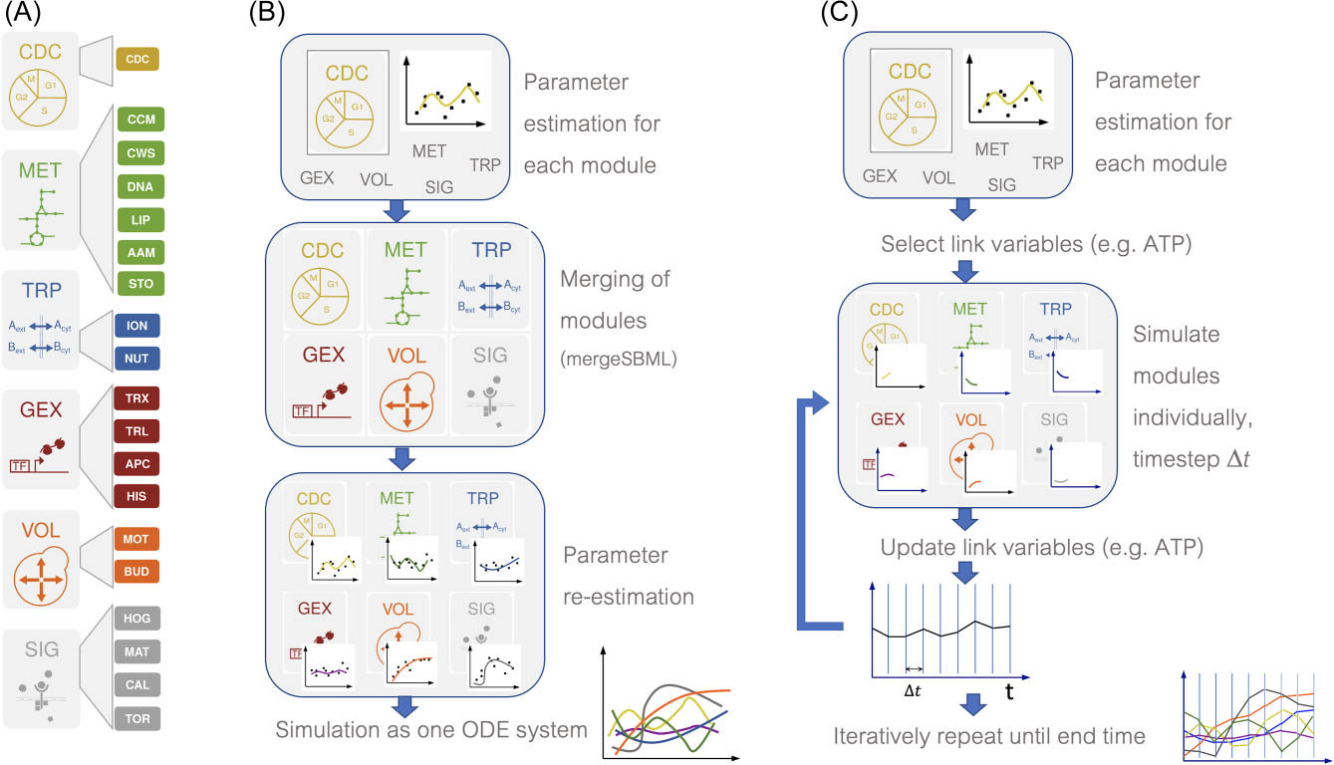
Modular approach to WCM. (A) Modules of the Yeast Cell Model: cell division cycle (CDC), metabolism [MET, containing central carbon metabolism (CCM), cell wall synthesis (CWS), DNA synthesis (DNA), lipid metabolism (LIP), amino acid metabolism (AAM), and storage (STO)], transport [TRP, comprising ion (ION) and nutrient (NUT) transport], gene expression [GEX, with transcription (TRX), translation (TRL), assembly of protein complexes (APC), and histone activity (HIS)], volume changes [VOL, for mother (MOT) and bud (BUD)], and signaling (SIG, including the high osmolarity glycerol (HOG), pheromone (MAT), Ca-calcineurin (CAL), and TOR (TOR) pathways)]. (B) Schematic of the merging approach: parameters are estimated for module separately with other modules reduced to critical information, e.g. just the relevant volume. Then modules are merged to one large ODE model, parameters are readjusted taking now the dynamics in other modules into account. Eventually, the whole WCM is simulated as one ODE system. (C) Schematic of consolidation approach: again, parameters are estimated for each module. In the simulation process, each module is simulated separately with its own algorithm for a given time step *Δt*. It takes given values of link variables as input, changes them during the simulation and provides them after *Δt* as output. All link variables are updated based on outputs of all modules. The process is iteratively repeated until the predefined end time.

A WCM consists of the computational description of a large amount of different cellular processes that relate to different cellular locations, different orders of magnitude of number of involved molecules, and different aspects considered to be of importance to the modeler or model user. There are, at least, two alternative ways of coping with this complexity: one can describe all processes with the same formalism, e.g. ordinary differential equations, or one can use the best suitable computational formalism for each process, see Fig. 4 (Hahn 2020). Both approaches come with their own advantages and disadvantages.

Figure 4 gives an overview of the approach. In its panel (A) it introduces the components of the Yeast Cell Model (YCM): the model is structured into distinct modules to represent various cellular processes:

- Metabolic module (MET): this module describes the biochemical reactions involved in cellular metabolism, including pathways for the synthesis and breakdown of metabolites such as sugars, amino acids, and nucleotides. It encompasses central carbon metabolism (CCM), cell wall synthesis (CWS), DNA synthesis (DNA), lipid metabolism (LIP), amino acid metabolism (AAM), and storage (STO).
- Gene expression module (GEX): this module models the regulatory networks that control gene expression, including transcription factors, regulatory proteins, and regulatory sequences in DNA. It covers transcription (TRX), translation (TRL), assembly of protein complexes (APC), and histone activity (HIS).
- Cell division cycle (CDC): this module represents the processes involved in cell growth and division, including DNA replication, chromosome segregation, and cell wall synthesis.
- Transport module (TRP): this module describes the movement of molecules across the cell membrane, including passive diffusion, active transport, and facilitated diffusion. It includes ion (ION) and nutrient (NUT) transport.
- Signaling module (SIG): this module models cellular signaling pathways, including receptor–ligand interactions, second messenger signaling, and signal transduction cascades. It includes pathways such as the high osmolarity glycerol (HOG), pheromone (MAT), Ca-calcineurin (CAL), and TOR pathways.
- Growth module (VOL): this module captures the requirements for an increase in cell volume as well as a description of the changes of volume and surface for both the mother (MOT) and bud (BUD).

Figure 4(B) provides an overview of the Merging Approach: in this approach, each module is initially analyzed separately with other modules simplified to critical information, such as relevant volume. Subsequently, these modules are merged into a unified Ordinary Differential Equation (ODE) model. Parameters are then recalibrated considering the interplay between dynamics in different modules. Eventually, the entire WCM is simulated as a single ODE system. Fig. 4(C) gives an outline of the Consolidation Approach in the context of the YCM, which involves several steps: (i) parameter estimation for each module: first, parameters are estimated for each individual module within the model. These parameters define the behavior and characteristics of each module, allowing them to function autonomously. (ii) Simulation Process for each module: each module operates independently during the simulation process. Using its own specific algorithm and the parameters determined in the first step, each module simulates its behavior over a given time step (*Δt*). (iii) Input and output handling: during simulation, each module takes input from specified link variables. These input values may include information from other modules or external factors. The module then processes this input, updates its internal state, and generates output variables. (iv) Updating link variables: after completing the simulation for a time step (*Δt*), the module outputs the revised variables. These updated variables may affect the behavior of other modules or influence the overall system dynamics. All link variables are then updated based on the outputs from all modules. (v) Iterative process: the simulation process iterates over multiple time steps until reaching a predefined end time. At each iteration, modules continue to interact with each other by exchanging information through link variables. This iterative approach allows the system to evolve dynamically over time. By following this consolidation approach, the YCM can capture the complex interactions and dynamics between different cellular processes while maintaining modularity and computational efficiency.

The formulation of a WCM as set of ordinary differential equations is potentially laborious, though achievable. If the model is formulated in SBML, semanticSBML is a tool intended for merging models of individual subprocesses (Krause et al. 2010). Merging has the advantage that even large systems of ODEs can nowadays be solved computationally in a reliable manner. It also allows smooth integration of different cellular functions into the same concept without overpronouncing the links between potentially defined modules (such as between metabolism providing ATP and amino acids and translation using ATP and amino acids). However, a disadvantage is that, given the complexity of a WCM and the different scales of times and abundances, it is unforeseeable in the near future that we will have parameter estimation tools available that will allow us to estimate model parameters from a data collection in one go. Since the WCM has a large number of parameters, most of them unknown, it is challenging that the state space, i.e. the range of values different model compounds can assume, is very large and the required repetitive simulations are computationally demanding.

Thus, we need to develop strategies to cope with this problem. One potential solution is to divide the full set of processes into a number of subsets or modules. In the context of whole-cell modeling, a module refers to a distinct component or subsystem within the overall model that represents a specific aspect of cellular function. Modules can be thought of as building blocks that are combined to form a comprehensive model of the entire cell. Each module typically focuses on modeling a particular set of molecular interactions or biological processes.

Each module may be represented using different mathematical and computational techniques, depending on the specific details of the processes being modeled. These modules are then integrated into a cohesive whole-cell model that captures the interactions and dynamics of the entire cell.

The boundaries and links of the subsets must be clearly defined. Then, one develops a coarse description of each subset, potentially guided by steady state assumption or other easy-to-obtain information. The union of all but one subset then serves as context for the remaining subset, providing it with boundary conditions and fluxes over its borders.

An alternative to the merging of all modules into one comprehensive ODE model is the consolidation approach. Such an approach was, e.g. used for the WCM for *M. genitalium* (Karr et al. 2012) as well for our YCM (Hahn 2020). Here again, models have to be developed for each module or cellular function. But these models can come with their own formalism, e.g. an ODE model for metabolism, a stochastic agent-based model for transcription and translation, and a stochastic model employing the Gillespie algorithm, i.e. a computational method used to simulate the time evolution of stochastic biochemical systems, for the cell cycle.

The modules share link variables, i.e. those variables that are changed in more than one of these modules and, thereby, connect these modules. Typical link variables are energy equivalents such as ATP and ADP or NAD and its derivatives, gene expression levels, signaling molecule activities, or the volumes of the cell and the compartments. Since they are changed in several modules, they serve as a communication channel and allow a flow of information between these modules. Their dynamics can be very complex, because it is influenced by the different modules.

Independent of the choice of approach to link the modules: a connection between the experimental data and the models or modules requires to estimate the model parameters from those experimental data. This is, according to our experience, frequently a challenging task for large and complex systems. Fortunately, by now there is a series of tools available to ease that task, e.g. parameter estimators with COPASI (Mendes et al. 2009) or Tellurium (Choi et al. 2018), MATLAB-related estimation programs such D2D (Raue et al. 2015), or estimators for Python programs (Schalte et al. 2023). For WCM, it is important to take care that parameters used in different modules are not conflicting, which potentially can be achieved by parameter re-estimation for the full model.

In summary, by breaking down the complexity of cellular systems into modular components, researchers can develop more manageable models and gain insights into the individual mechanisms underlying cellular function. Additionally, modular modeling approaches allow for flexibility and scalability, enabling us to add or modify modules as new experimental data becomes available or as the model is extended to study different organisms or cellular processes.

### Toward a comprehensive YCM

The yeast community has created a comprehensive series of models of different cellular processes that can serve as modules for a whole-cell model of yeast (YCM), though they also stand in their own right to integrate data and explain biological observations. These are models for cell cycle in different detail and formalisms such as (Chen et al. 2000, 2004, Barberis et al. 2007, Zhang et al. 2011, Spiesser et al. 2012, Palumbo et al. 2016, Münzner et al. 2019, Adler et al. 2022). Other models describe volume changes of mother and bud (Altenburg et al. 2019). DNA replication has also gotten attention (e.g. Brümmer et al. 2010, Spiesser et al. 2010). Signaling pathways have attracted a multitude of modeling efforts, either in their own right (Yi et al. 2003, Kofahl and Klipp 2004, Sackmann et al. 2006, Schaber et al. 2006, 2012, Waltermann and Klipp 2010, Thomson et al. 2011, Lubitz et al. 2015, Stojanovski et al. 2017, Dunayevich et al. 2018, Pomeroy et al. 2021) or as models combining signaling with complex regulation processes (Klipp et al. 2005, Adrover et al. 2011). Metabolism has experienced a series of comprehensive reconstructions, often community-based (herrgaard et al. 2008, Lu et al. 2019), however, there are also dynamic detailed models for different parts of metabolism including glycolysis (Rizzi et al. 1997, Hynne et al. 2001, Lao-Martil et al. 2023, Van Heerden et al. 2014, Smallbone et al. 2013) or lipid metabolism (Schützhold et al. 2016). Ion transport has been described for single cells (e.g. Kahm et al. 2012, Gerber et al. 2016) Gene expression is frequently analyzed in statistic models, but there are also approaches for dynamic models of transcription (e.g. Amoussouvi et al. 2018) and translation (e.g. Seeger et al. 2023).

Unfortunately, the list of mentioned works cannot be complete, since there are so many, which underlines the deep interest in the dynamics of the yeast *S. cerevisiae* as model organism.

Yet, the full establishment of a YCM that describes a single cell and is comprehensive, dynamic and covers a whole cell cycle is—to our best knowledge—still future. This can have a series of reasons. First, as the above referenced literature may indicate, the coverage of cellular processes with detailed dynamic mathematical models is not equally dense. Some pathways or networks have attracted much more modeling efforts than others, e.g. glycolysis more than amino acid synthesis or cell wall synthesis or the pheromone pathway more than the starvation response pathway. Second, experimental data with temporal resolution over one cell cycle that can be interpreted for average single cells is still rare. This is in part due to the fact, that cell-cycle resolution on a population level requires synchronization, which always interferes with the cell’s normal processes. Mostly used synchronization by adding and removing again the pheromone alpha-factor activates the pheromone pathway and leaves behind cells that are much larger than average (because they have grown during pheromone treatment) and, therefore, have shorter G1 phases than an average population. Synchronization by elutriation provides low yields of newborn small G1 cells, but also exerts stress on those cells. While time-resolved and quantitative data are invaluable for systems biology modeling, it is important to acknowledge that such data may not always be readily available, even not in the YCMDB, or comprehensive. Systematically measuring time-resolved data at different conditions and perturbations can indeed enhance the reconstruction of a YCM. The systematic reconstruction of a YCM may involve iterative refinement and integration of diverse data sources to capture the complexity of biological systems accurately. Eventually, the creation of a YCM is a complex effort that would certainly profit from a concerted action of interested parties.

### Such a consensus YCM could be very valuable for many purposes such as

- systematic integration of high-throughput and small-scale experimental data;
- methodical testing of hypothesis of the role of individual compounds and their interactions;
- prediction for cellular behavior after simple or complex perturbations, but also in different media or other external conditions;
- elucidation of complex, often long-ranging interactions that are not experimentally accessible;
- context for new findings in yeast cell biology;
- information base for microbial cell factory projects;
- blueprint for a eukaryotic WCM.

**Figure 5.**
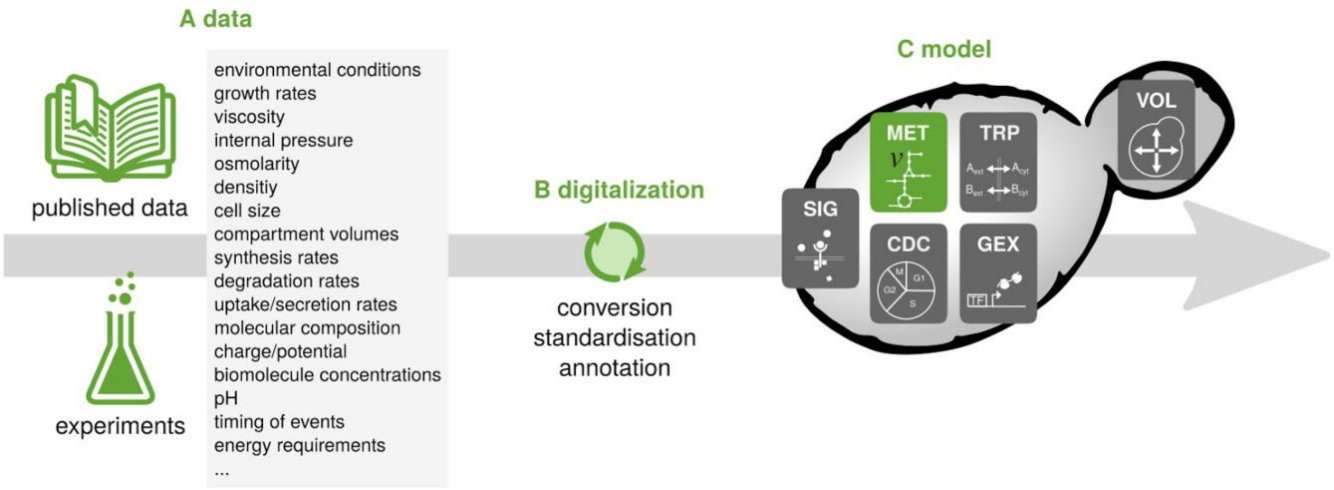
Information flow for the creation of a YCM. (A) Literature and experimental results hold information on a multitude of biological processes and interactions. However, this data is highly condition dependent and stored in nonstandardized ways. (B) To reproducibly use the data, understand their connection, and judge their consistency and information content, several nontrivial digitalization steps are required. (C) The curated data can then be consistently and formally analyzed, e.g. in mathematical models, to foster the understanding of underlying biological processes, but also to reveal knowledge gaps. Eventually, models of whole cells could be simulated to understand the complex interwiring of cellular processes.

Such an integrated model will allow to answer many biologically relevant questions on a much more solid foundation than the individual models for selected processes that cannot capture the full context of the processes under investigation. This requires a systematic and coordinated flow of information from literature and dedicated experiments via digitalization into the YCM (Fig. 5).

## Supplementary Material

foae011_Supplemental_File
