# Supplementary material for: Data integration strategies for whole-cell modeling
Source: FEMS Yeast Res. 2024 Mar 27;24:foae011. doi: 10.1093/femsyr/foae011 (PMC11042497; doi:10.1093/femsyr/foae011)
Supplement: foae011_Supplemental_File [file foae011_supplemental_file.docx]

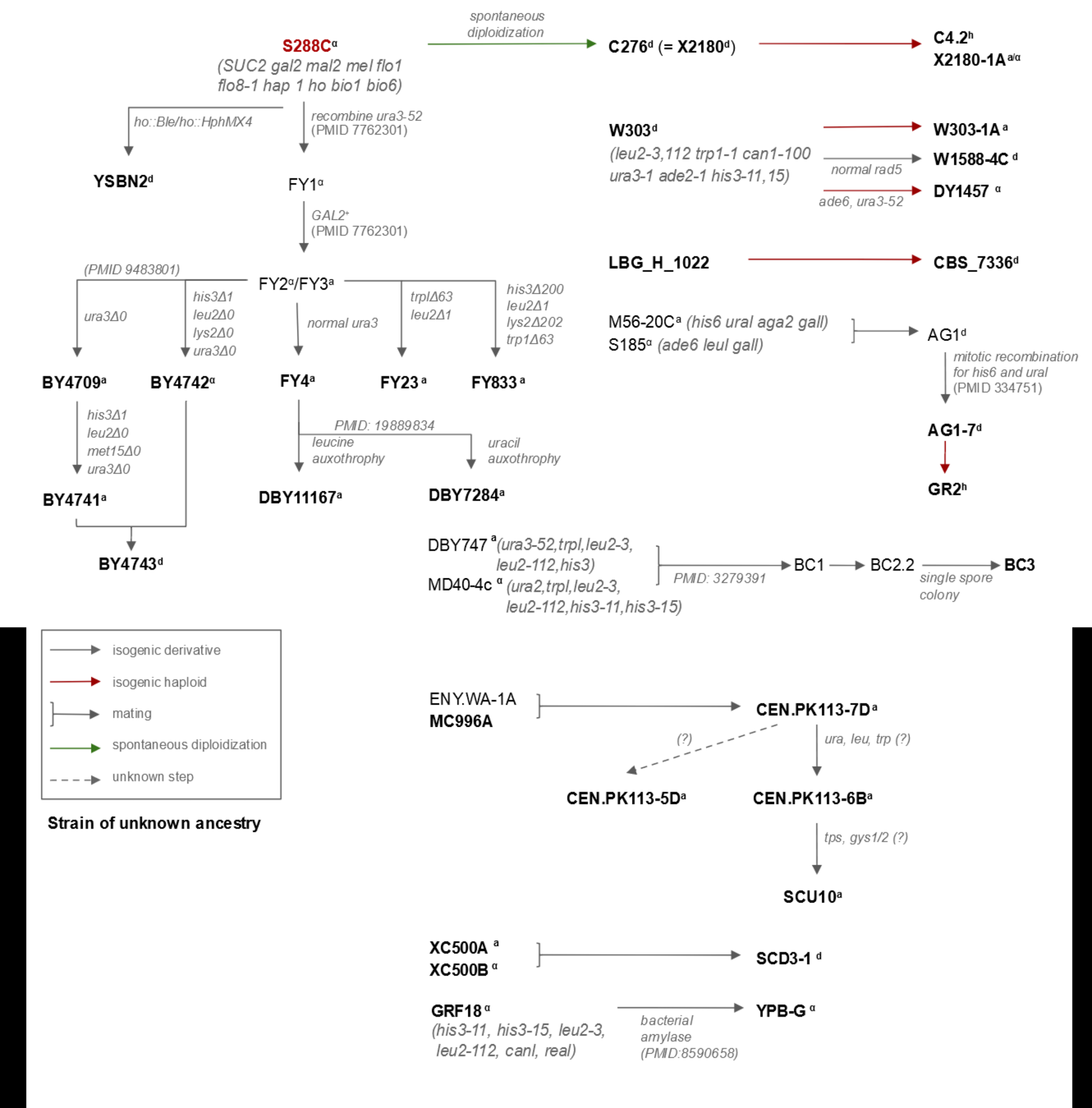


**Supplementary Figure S1:** Evolutionary dependence of yeast strains, for which data is present in the Yeast Cell Model Data Base (YCMDB)
